# Au Nanoshell-Based Lateral Flow Immunoassay for Colorimetric and Photothermal Dual-Mode Detection of Interleukin-6

**DOI:** 10.3390/molecules29153683

**Published:** 2024-08-03

**Authors:** Congying Wen, Yue Dou, Yao Liu, Xuan Jiang, Xiaomei Tu, Ruiqiao Zhang

**Affiliations:** 1 College of Chemistry and Chemical Engineering, China University of Petroleum (East China), Qingdao 266580, China; douy126@126.com (Y.D.); 17663987932@163.com (Y.L.); 17660637078@163.com (X.J.); 2203040221@s.upc.edu.cn (X.T.); 2 Qingdao Academy of Agricultural Sciences, Qingdao 266100, China

**Keywords:** Au_shell_ nanoparticles, Interleukin-6, lateral flow immunoassay, colorimetric detection, photothermal detection

## Abstract

Interleukin-6 (IL-6) detection and monitoring are of great significance for evaluating the progression of many diseases and their therapeutic efficacy. Lateral flow immunoassay (LFIA) is one of the most promising point-of-care testing (POCT) methods, yet suffers from low sensitivity and poor quantitative ability, which greatly limits its application in IL-6 detection. Hence, in this work, we integrated Au_shell_ nanoparticles (NPs) as new LFIA reporters and achieved the colorimetric and photothermal dual-mode detection of IL-6. Au_shell_ NPs were conveniently prepared using a galvanic exchange process. By controlling the shell thickness, their localized surface plasmon resonance (LSPR) peak was easily tuned to near-infrared (NIR) range, which matched well with the NIR irradiation light. Thus, the Au_shell_ NPs were endowed with good photothermal effect. Au_shell_ NPs were then modified with IL-6 detection antibody to construct Au_shell_ probes. In the LFIA detection, the Au_shell_ probes were combined with IL-6, which were further captured by the capture IL-6 antibody on the test line of the strip, forming a colored band. By observation with naked eyes, the colorimetric qualitative detection of IL-6 was achieved with limit of 5 ng/mL. By measuring the temperature rise of the test line with a portable infrared thermal camera, the photothermal quantitative detection of IL-6 was performed from 1~1000 ng/mL. The photothermal detection limit reached 0.3 ng/mL, which was reduced by nearly 20 times compared with naked-eye detection. Therefore, this Au_shell_-based LFIA efficiently improved the sensitivity and quantitative ability of commercial colloidal gold LFIA. Furthermore, this method showed good specificity, and kept the advantages of convenience, speed, cost-effectiveness, and portability. Therefore, this Au_shell_-based LFIA exhibits practical application potential in IL-6 POCT detection.

## 1. Introduction

Interleukin-6 (IL-6) is an important pleiotropic cytokine, which is closely related to immune regulation, inflammation, hematopoiesis, oncogenesis, and so on [1,2]. The IL-6 level increases in many diseases, such as infectious diseases, immunological diseases, multiple sclerosis, Alzheimer disease, and various cancers [3,4,5]. The increase degree is positively correlated to the severity of disease [6,7,8]. Now, IL-6 detection and monitoring have been used to evaluate the progression of many diseases and their therapeutic efficacy. Currently, clinical detection methods including enzyme linked immunoassay, radioimmunoassay, and chemiluminescent immunoassay have high sensitivity and accuracy, yet suffer from the drawbacks of time-consuming and complicated manipulation, sophisticated equipment, and a high dependence on professional staff and workplaces [9,10,11]. With the increasing demand for point-of-care testing (POCT) and home self-testing, it is very necessary to develop simple, rapid, sensitive, and low-cost methods for detecting IL-6.

Lateral flow immunoassay (LFIA) possesses the characteristics of convenience, portability, rapidity, and cost-effectiveness, which does not require complicated instruments or skilled staff [12,13,14]. Thus, LFIA shows promising advantages in POCT. As a successful example, pregnancy test strips have been applied by numerous people to achieve rapid home self-test, avoiding the trouble of going to hospital. However, nearly all of the commercial LFIAs use gold nanoparticles (NPs) as reporters. These colloidal gold strips can only give a “yes or no” result and cannot achieve accurate quantification. Moreover, due to the limited localized surface plasmon resonance (LSPR) effect of gold NPs, colloidal gold strips exhibit low sensitivity [15,16,17]. These limitations greatly restrict their application to IL-6 detection.

Recently, researchers have made various efforts to improve the performance of traditional LFIA. On the one hand, researchers have developed various signal amplification methods to enhance the colorimetric signals on the test zones, such as enzyme-based color enhancement and in situ noble metal shell growth [18,19,20,21]. These methods indeed increase the sensitivity for naked-eye determination. However, the additional amplification steps usually make the manipulation more complex and time-consuming. On the other hand, some researchers have modified the LFIA and developed a vertical flow assay (VFA) [22,23]. VFA can process a greater volume of samples or high-viscosity samples, thus improving the detection sensitivity. But VFAs usually have complicated operation principles, making them unable to compete with LFIAs in practice [24]. Compared with the first two attempts, integrating new nanomaterials with better signals into a LFIA to replace gold NPs may be a more convenient and effective method to improve the limitations of traditional LFIAs. Currently, quantum dots, surface-enhanced Raman scattering nanomaterials, magnetic nanomaterials, photothermal nanomaterials, and so on have been used in LFIAs, achieving higher sensitivity and accurate quantification [25,26,27,28,29]. Among all these new labels, photothermal nanomaterials can convert light into heat, producing temperature rises as signals, which do not need a complex or expensive readout instrument [30,31,32]. Moreover, the development of highly sensitive infrared cameras which can distinguish ±0.025 °C makes photothermal sensing usually have a high resolution [33,34]. Furthermore, the temperature signal is hardly interfered by the matrix color, endowing photothermal detection with a strong anti-interference ability [34,35]. Thus, photothermal nanomaterials have become one of the most promising LFIA reporters. For example, Zhang et al. constructed a black phosphorus-Au nanocomposite to function as a LFIA signal probe and achieved an ultrasensitive detection of 17β-estradiol and mycotoxin zearalenone with limits of 50 pg/mL and 10 pg/mL, respectively, which was nearly 100-fold more sensitive than a visual LFIA [36,37]. As another example, Hao et al. prepared ReSe_2_ nanosheets using liquid exfoliation, and with them a photothermal LFIA detection of human anti-SARS-CoV-2 S protein antibodies was performed with a detection limit of 0.86 ng/mL, which was 108-fold lower than that of the colorimetric signal [38]. In recent years, our group also has been dedicated to developing photothermal LFIAs. For example, we designed dumbbell-like Au-Fe_3_O_4_ NPs with a seed-mediated growth method, and realized the sensitive photothermal detection of *Salmonella* and SARS-CoV-2 [39,40]. The above studies show that integrating photothermal nanomaterials as new reporters indeed improves the detection sensitivity of a traditional LFIA. However, most of the photothermal nanomaterials usually comprise two parts: the colorimetric part such as Au and the photothermal part such as black phosphorus. Thus, the preparation procedures of most of the photothermal nanomaterials were much more complicated than gold NPs, limiting their practical application. Therefore, it is necessary to develop a photothermal LFIA reporter with a simple and low-cost synthetic method.

At present, near-infrared (NIR) irradiation light is most commonly used in photothermal detection, which can greatly reduce the background signal. Thus, to obtain a good photothermal effect, it is very necessary to tune the nanomaterials’ LSPR absorption peak to the NIR range. As reported, the gold nanoshells’ LSPR peak can be easily tuned from the visible to the NIR range just by controlling the shell thickness [41,42]. Hence, in this work, we used a galvanic exchange process to prepare NIR gold nanoshells, and applied them to a dual-mode LFIA detection of IL-6. The gold nanoshells possessed a good colorimetric signal and photothermal effect without combining other photothermal nanomaterials. Furthermore, the synthesis step could be easily performed. Using gold nanoshells as LFIA reporters, by observation with naked eyes, the colorimetric qualitative detection of IL-6 was achieved with a limit of 5 ng/mL. By measuring the temperature change in the test lines (T lines) after laser irradiation, the photothermal quantitative detection of IL-6 was performed from 1~1000 ng/mL. Under optimal conditions, the photothermal detection limit reached 0.3 ng/mL, which was reduced by nearly 20 times compared with that of naked-eye detection. Therefore, this Au_shell_-based LFIA efficiently improved the sensitivity and quantitative ability of commercial colloidal gold LFIA, showing a practical application value.

## 2. Results and Discussion

### 2.1. Principle of the Au_shell_-Based LFIA for Detecting IL-6

**Scheme 1:**
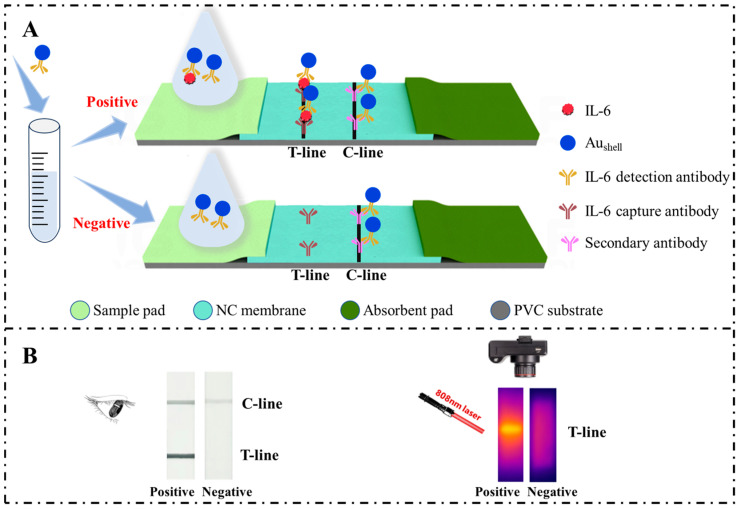
(**A**) Schematic diagram for the principle of the Au_shell_-based LFIA for detecting IL-6. (**B**) Typical naked-eye observation and photothermal results of positive and negative samples.

As illustrated in Scheme 1A, based on the antibody–antigen reaction, the Au_shell_ probes combine with IL-6 and then are loaded on the strip; these are then captured by the IL-6 capture antibody on the T line, forming a colored band. Free Au_shell_ probes migrate further and bind with the goat anti-mouse secondary antibody on the control line (C line), forming a second colored band. Thus, positive samples produce two colored bands, while in negative samples, no Au_shell_ probe-IL-6 complexes can accumulate in the T line, and only the Au_shell_ probes are captured by the C line. Thus, negative samples only produce a colored C line. In cases where the C line does not show any color, it means that the antibodies on the probes or the strips are inactivated, and the results are invalid. By observing the color bands with naked eyes, the colorimetric qualitative detection of IL-6 is achieved. By measuring the temperature change in the T lines after laser irradiation, the photothermal quantitative detection of IL-6 is performed. The typical detection results of the positive and negative samples are illustrated in Scheme 1B. It can be seen that the positive sample produced two obvious blue-black bands, while the negative sample only showed one blue-black control band. In the photothermal images, the T line of the positive sample showed an obvious high temperature, while the T line of negative sample did not show any obvious high-temperature zones.

### 2.2. Characterization of Au_shell_ and Au_shell_ Probes

**Figure 1:**
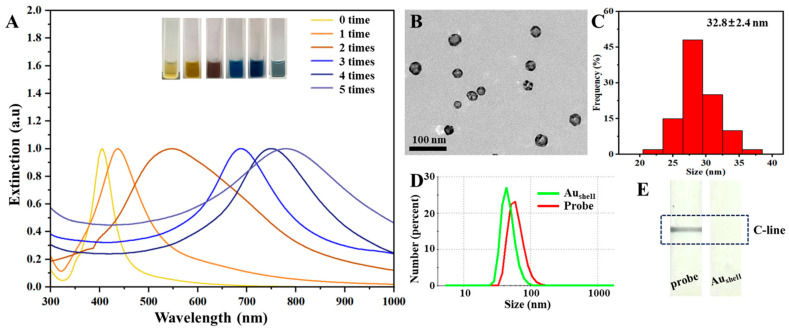
(**A**) UV-Vis spectra and corresponding photos of Au_shell_ NPs with different times of chloroauric acid additions. (**B**) TEM image of Au_shell_ NPs. (**C**) Size distribution of Au_shell_ NPs. (**D**) Hydrodynamic diameter distributions of Au_shell_ NPs and Au_shell_ probes. (**E**) Photos of the IL-6 strips loaded with Au_shell_ NPs and Au_shell_ probes.

To obtain the best photothermal effect, the LSPR absorption peak of the Au_shell_ NPs should match the 808 nm irradiation laser. With increasing the amount of chloroauric acid, the etching enhanced, and the Au_shell_ absorption peak red shifted. As shown in Figure 1A, after five additions of chloroauric acid, the absorption peak reached 779 nm. Further increasing the chloroauric acid amount would make the peak shift further to longer wavelength. However, excessive chloroauric acid would destroy the Au_shell_ structure, resulting in gold rings, which would decrease the stability [42]. Hence, through the comprehensive consideration of the photothermal effect and stability, five additions of chloroauric acid were chosen. The obtained Au_shell_ showed satisfactory dispersibility with a diameter of 32.8 nm (Figure 1B,C). After modification with IL-6 detection antibody, their hydrodynamic diameter increased from 46.96 nm to 62.08 nm (Figure 1D). When the Au_shell_ and Au_shell_ probes were loaded on the IL-6 strips, only the Au_shell_ probes could produce a colored C line, while Au_shell_ NPs could not accumulate on the C line (Figure 1E). The above results suggested that the IL-6 detection antibody was successfully conjugated with the Au_shell_ NPs and retained a good bioactivity.

### 2.3. Investigation of the Optimal Detection Conditions

**Figure 2:**
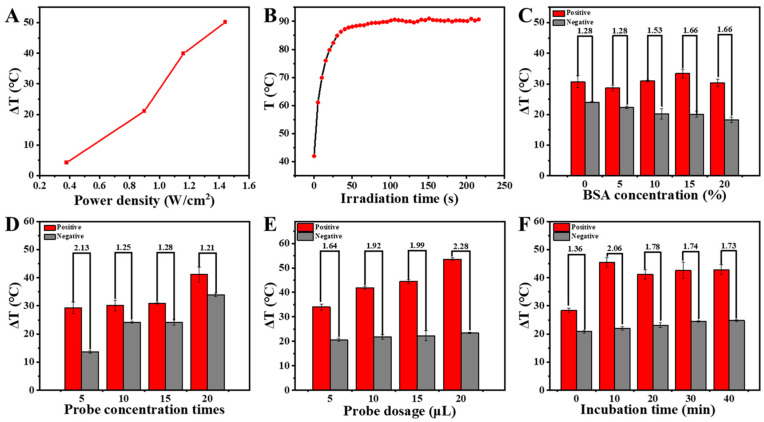
Photothermal signals obtained at different irradiation power densities (**A**), irradiation times (**B**), BSA concentrations for blocking (**C**), Au_shell_ probe concentration times (**D**), Au_shell_ probe amounts (**E**), and incubation times (**F**). The numbers above the columns are the signal-to-noise ratios. Error bars = ±SD (n = 3).

To achieve the best analytical performance, several important detection conditions were optimized, such as irradiation power density and time, bovine serum albumin (BSA) concentration for blocking, Au_shell_ probe concentration, amount of Au_shell_ probes, and incubation time. As shown in Figure 2A, with an increase in the irradiation power density, ΔT increased. When 1.44 W/cm^2^ was used, ΔT reached more than 50 °C, and correspondingly, the temperature attained from the tested strip reached as high as 99.5 °C. In consideration of protecting the strips, 1.16 W/cm^2^ was chosen as the optimal power density. Similarly, with increasing irradiation time, the temperature of the tested strip increased. At 100 s, the temperature reached a stable and maximum value (Figure 2B), which was chosen as the optimal irradiation time. For other conditions’ optimization, the condition which could obtain the highest ratio of the positive sample signal to the negative sample signal (signal-to-noise ratio) was selected. According to the experiment results (Figure 2C–F), 15% BSA, Au_shell_ probes concentrated by five times, 20 μL of Au_shell_ probes, and 10 min incubation were finally chosen for further detection.

### 2.4. Colorimetric Qualitative Detection of IL-6 with the Au_shell_-Based LFIA

**Figure 3:**
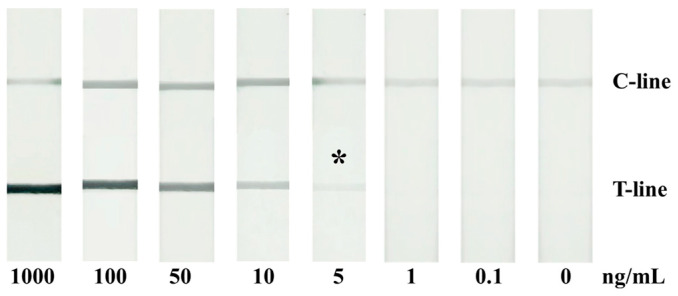
Photos of LFIA strips for detection of different concentrations of IL-6. The asterisk represents the naked-eye limit of detection (LOD).

Under optimal detection conditions, we investigated the colorimetric qualitative detection performance of the Au_shell_-based LFIA. As shown in Figure 3, the negative sample only produced one colored C line. When the IL-6 concentration reached 5 ng/mL, the T line began to exhibit a visible blue-black color, and with the IL-6 concentration further increasing, the color of the T line became darker. This was because more IL-6 would produce more IL-6-Au_shell_ complexes, and hence more Au_shell_ would accumulate on the T line, thus resulting in a darker color. By observing the T line color, the colorimetric qualitative detection of IL-6 could be achieved with a naked-eye detection limit of 5 ng/mL.

### 2.5. Photothermal Quantitative Detection of IL-6 with the Au_shell_-Based LFIA

**Figure 4:**
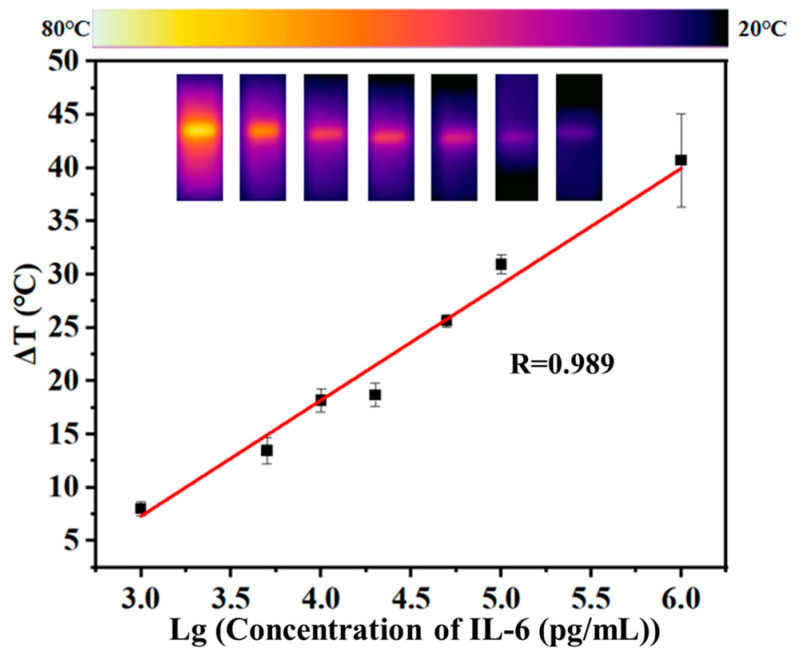
Linear relationship of photothermal signal (ΔT) versus IL-6 concentration. The corresponding photothermal images are shown in the inset. Error bars = ±SD (n = 3).

Under optimal detection conditions, we further investigated the photothermal quantitative detection performance of the Au_shell_-based LFIA. As shown in Figure 4, with the increase in IL-6 concentration, the ΔT of the T line increased. In the IL-6 concentration range of 1~1000 ng/mL, the ΔT was clearly linear with the common logarithm of the IL-6 concentration. The LOD was calculated to be 0.3 ng/mL with the standard IUPAC method (LOD signal = ΔT_blank_ + 3SD_blank_, where ΔT_blank_ is the average ΔT obtained from 11 blank samples, and SD_blank_ is their standard deviation). It could be seen that the photothermal detection not only achieved the quantification of IL-6, but it also enhanced the detection sensitivity. The photothermal LOD was reduced by nearly 20 times compared with that of naked-eye detection.

### 2.6. Specificity Investigation

**Figure 5:**
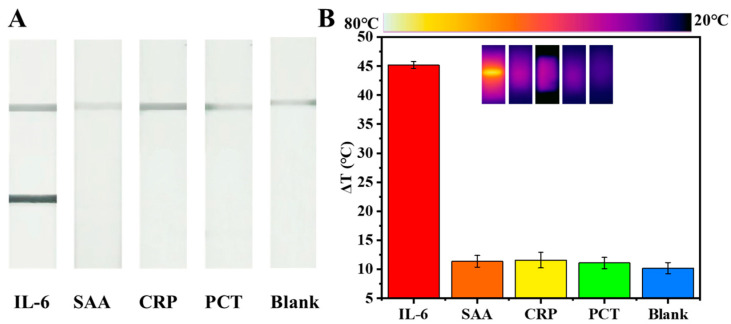
Results from detection of different cytokines with the Au_shell_-based LFIA. (**A**) Photos of the test strips. (**B**) ΔT histograms and photothermal images of the test strips.

To investigate the specificity of the Au_shell_-based LFIA, several common cytokines with 10 times higher concentrations than IL-6 were detected. As shown in Figure 5A, IL-6 groups showed obvious colored T line and C line, while in other cytokine groups, the T lines were invisible, and only the C line exhibited color. When measuring the photothermal signals, the IL-6 groups showed high ΔT, while the ΔTs from other cytokine groups were very low, which were nearly the same as those from the blank groups (Figure 5B). Hence, this LFIA had good specificity.

## 3. Experimental Section

### 3.1. Reagents and Instruments

Polyvinylpyrrolidone, trisodium citrate, potassium carbonate, sucrose, sodium borohydride, chloroauric acid, Tween-20, and silver nitrate were purchased from Macklin Biochemical Co., Ltd. (Shanghai, China). Bovine serum albumin (BSA) was obtained from Saiguo biotech Co., Ltd. (Guangzhou, China). PBS (pH = 7.4) was purchased from Labgic Technology Co., Ltd. (Beijing, China). IL-6, IL-6 detection antibody, IL-6 capture antibody, goat anti-mouse secondary antibody, serum amyloid protein A (SAA), C-reactive protein (CRP), and procalcitonin (PCT) were supplied by Henderson Biotechnology Co., Ltd. (Qingdao, China). Nitrocellulose (NC) membrane (CN95), sample pad, absorbent pad, and polyvinyl chloride (PVC) substrate were purchased from Joey-biotech Co., Ltd. (Shanghai, China). A UV-Vis spectrophotometer (UV-2450, Shimadzu, Kyoto, Japan) was used to measure absorption spectra. An electron microscope (JEM 1400, JEOL, Tokyo, Japan) was used to take transmission electron microscopy (TEM) images. A precision sprayer (HGS510, AUTOKUN, Hangzhou, China) and high-speed guillotine cutter (HGS210, AUTOKUN, Hangzhou, China) were used to spray and divide test strips. A Zetasizer Nano ZS instrument (Malvern, Britain) was used to measure hydrodynamic diameter. An infrared thermal imager (FOTRIC 226s, Shanghai, China) and an 808 laser (FU808AD2000-F34, Shenzhen, China) were used to measure photothermal signals.

### 3.2. Preparation of Au Nanoshells

Au nanoshells were prepared via a nanoscale galvanic exchange process according to the previous work [42]. A total of 20 mL of 1% trisodium citrate and 75 mL of ultra-pure water were mixed and heated to 70 °C. After 15 min, 1.7 mL of 1% silver nitrate was added and 2 mL of 1% sodium borohydride was quickly injected for 1 h reaction to obtain Ag seeds. Then, 20 mL of Ag seeds and 3.4 mL of 1% silver nitrate were added to the boiling mixture, containing 4 mL of 1% trisodium citrate and 150 mL of ultra-pure water. The reaction lasted for 1 h. Afterwards, 4 mL of 1% trisodium citrate and 3.4 mL of 1% silver nitrate were added to the above mixture for another 1 h reaction. Trisodium citrate and silver nitrate were added and reacted once again. After cooling down to room temperature, the above solution was irradiated with 254 nm ultraviolet light for 40 min, and Ag NPs were obtained. Then, 5 mL of Ag NPs and 45 mL of polyvinylpyrrolidone (1 mg/mL) were mixed and heated to 100 °C. After 15 min, chloroauric acid was added drop by drop at a rate of 1 mL/min every 2 min. After 5 times, the reaction was terminated by ice water, and Au nanoshells were obtained.

### 3.3. Construction of Au_shell_ Probes Targeting IL-6

Au nanoshells were first concentrated by centrifugation. Then, 1 mL of the concentrated Au nanoshells was added with 0.1 mol/L potassium carbonate to adjust pH to 8~9, and 29.4 μL of IL-6 detection antibody (1 mg/mL) was added for 45 min reaction with shaking. After that, the Au_shell_ probes were centrifuged and resuspended in BSA solution for 30 min blocking. Finally, the Au_shell_ probes were washed 3 times using centrifugation and resuspended in 1 mL of pH = 7.4 PBS containing 1% BSA, 0.5% tween-20, and 1% sucrose for further use.

### 3.4. Fabrication of IL-6 LFIA Strips

As illustrated in Scheme 1, the strip was composed of PVC substrate, sample pad, NC membrane, and absorbent pad. For fabrication, NC membrane was first pasted on middle of the PVC substrate. Then, 1.7 cm width absorbent pad was pasted on the right with ca. 0.3 cm overlap, and 2.2 cm width sample pad was pasted on the left with ca. 0.3 cm overlap. T line was sprayed with IL-6 capture antibody (1 mg/mL) and C line was sprayed with goat anti-mouse secondary antibody (1 mg/mL), both at a rate of 1 μL/cm. Finally, the strips were dried at 37 °C and then cut into 3 mm width for use.

### 3.5. Detection of IL-6 with the Au_shell_-Based LFIA

Typically, 20 μL of sample solution were added with Au_shell_ probes, which were further added with PBS (pH = 7.4, containing 1% BSA, 0.5% tween-20, and 1% sucrose) to make the total volume reach 50 μL. The above mixture was added to a microplate. After incubation with gentle shaking, the sample pad of the LFIA strip was immersed in the solution. After 15 min, by observing the T line colors with naked eyes, colorimetric qualitative detection of IL-6 was achieved. By measuring temperature change (∆T) of T lines after 808 nm laser irradiation, photothermal quantitative detection of IL-6 was performed. To eliminate the impact of irradiation on the strip temperature, ∆T was defined as follows:

∆T = T_1_ − T_0_ (1)

where T_1_ and T_0_ are, respectively, the temperature of the highest temperature zone attained from the tested strip and unused strip after irradiation.

### 3.6. Optimization of the Detection Conditions

For irradiation power density optimization, the test line of one strip was irradiated, respectively, at 0.38, 0.9, 1.16, and 1.44 W/cm^2^, and the corresponding ∆T was measured for comparison. For the irradiation time optimization, the ∆Ts obtained at different irradiation times were recorded for comparison. For the optimization of BSA concentration for blocking, the Au_shell_ probe concentration, the amount of Au_shell_ probes, and the incubation time, a single factor variable method was performed. This involves only varying one condition while fixing other conditions, and the corresponding signal-to-noise ratio was calculated for comparison. Specifically, for BSA concentration optimization, 0%, 5%, 10%, 15%, and 20% BSA were, respectively, used to block the Au_shell_ probes, and applied to the IL-6 LFIA detection under the other fixed conditions (Au_shell_ probes by 10 times concentration, 10 μL of Au_shell_ probes, and 10 min incubation). For Au_shell_ probe concentration optimization, Au_shell_ probes with peak absorbance of ca. 0.7 were, respectively, concentrated by 5, 10, 15, 20 times and then used for IL-6 detection under the other fixed conditions (5% BSA, 10 μL of Au_shell_ probes, and 10 min incubation). For Au_shell_ probe amount optimization, 5, 10, 15, and 20 μL of Au_shell_ probes were, respectively, used under the other fixed conditions (5% BSA, Au_shell_ probes by 5 times concentration, and 10 min incubation). For incubation time optimization, 0, 10, 20, 30, and 40 min were, respectively, used under the other fixed conditions (5% BSA, Au_shell_ probes by 5 times concentration, and 10 μL of Au_shell_ probes).

## 4. Conclusions

In summary, we developed an Au nanoshell-based LFIA for the colorimetric and photothermal dual-mode detection of IL-6. The Au nanoshells were conveniently prepared by galvanic exchange process. Its LSPR peak could be easily tuned to the NIR range to match well with the NIR irradiation light. As a result, the Au nanoshells possessed a good colorimetric signal and a high photothermal effect without combining with other photothermal nanomaterials. We integrated the Au nanoshells as LFIA reporters and systematically optimized the related detection conditions. Under optimal conditions, the colorimetric qualitative detection of IL-6 could be achieved with a naked-eye LOD of 5 ng/mL, and the photothermal quantitative detection of IL-6 was performed from 1~1000 ng/mL with a LOD of 0.3 ng/mL, which was reduced by nearly 20 times compared with naked-eye detection. Therefore, the photothermal detection not only achieved the quantification of IL-6, but also enhanced the detection sensitivity. Furthermore, this Au nanoshell-based LFIA had good specificity, could be manipulated conveniently and rapidly, and did not need sophisticated readout instrument. Thus, this Au nanoshell-based LFIA efficiently improved the limitations of traditional LFIAs, and showed a practical application potential in IL-6 POCT detection.

## Data Availability

Data are contained within the article.

## References

[1] Kang S., Tanaka T., Narazaki M., Kishimoto T. (2019). Targeting Interleukin-6 Signaling in Clinic. Immunity.

[2] Cancelliere R., Di Tinno A., Di Lellis A.M., Contini G., Micheli L., Signori E. (2022). Cost-effective and disposable label-free voltammetric immunosensor for sensitive detection of interleukin-6. Biosens. Bioelectron..

[3] (2021). Interleukin-6 Receptor Antagonists in Critically Ill Patients with COVID-19. N. Engl. J. Med..

[4] Smolen J.S., Beaulieu A., Rubbert-Roth A., Ramos-Remus C., Rovensky J., Alecock E., Woodworth T., Alten R. (2008). Effect of interleukin-6 receptor inhibition with tocilizumab in patients with rheumatoid arthritis (OPTION study): A double-blind, placebo-controlled, randomised trial. Lancet.

[5] Kumari N., Dwarakanath B.S., Das A., Bhatt A.N. (2016). Role of interleukin-6 in cancer progression and therapeutic resistance. Tumor Biol..

[6] Dou C., Wu Z., Chen W., Yan H., Li D., You X.-Q., Chen Y.-S., Zhou C., Chen S., Zhuang P. (2024). Au-functionalized wrinkle graphene biosensor for ultrasensitive detection of Interleukin-6. Carbon.

[7] Russell C., Ward A.C., Vezza V., Hoskisson P., Alcorn D., Steenson D.P., Corrigan D.K. (2019). Development of a needle shaped microelectrode for electrochemical detection of the sepsis biomarker interleukin-6 (IL-6) in real time. Biosens. Bioelectron..

[8] Wang Y.-C., Lin S.-W., Wang I.J., Yang C.-Y., Hong C., Sun J.-R., Feng P.-H., Lee M.-H., Shen C.-F., Lee Y.-T. (2022). Interleukin-6 Test Strip Combined With a Spectrum-Based Optical Reader for Early Recognition of COVID-19 Patients With Risk of Respiratory Failure. Front. Bioeng. Biotechnol..

[9] Majdinasab M., Lamy de la Chapelle M., Marty J.L. (2023). Recent Progresses in Optical Biosensors for Interleukin 6 Detection. Biosensors.

[10] Fan G.-C., Ren X.-L., Zhu C., Zhang J.-R., Zhu J.-J. (2014). A new signal amplification strategy of photoelectrochemical immunoassay for highly sensitive interleukin-6 detection based on TiO2/CdS/CdSe dual co-sensitized structure. Biosens. Bioelectron..

[11] Shao Z.-H., Mo H.-L., Zhao X., Xie F., Zhao G. (2023). Atomic-precise Pt2Cu4 cluster-based fluorescent sensor for rapid interleukin-6 detection. Anal. Methods.

[12] Mak W.C., Beni V., Turner A.P.F. (2016). Lateral-Flow Technology: From Visual to Instrumental. TrAC-Trend. Anal. Chem..

[13] Boehringer H.R., O’Farrell B.J. (2022). Lateral Flow Assays in Infectious Disease Diagnosis. Clin. Chem..

[14] Sohrabi H., Majidi M.R., Khaki P., Jahanban-Esfahlan A., de la Guardia M., Mokhtarzadeh A. (2022). State of the art: Lateral flow assays toward the point-of-care foodborne pathogenic bacteria detection in food samples. Compr. Rev. Food Sci. Food Saf..

[15] Hu J., Zhang Z.L., Wen C.Y., Tang M., Wu L.L., Liu C., Zhu L., Pang D.W. (2016). Sensitive and Quantitative Detection of C-Reaction Protein Based on Immunofluorescent Nanospheres Coupled with Lateral Flow Test Strip. Anal. Chem..

[16] Omidfar K., Riahi F., Kashanian S. (2023). Lateral Flow Assay: A Summary of Recent Progress for Improving Assay Performance. Biosensors.

[17] Yin X., Liu S., Kukkar D., Wang J., Zhang D., Kim K.-H. (2024). Performance enhancement of the lateral flow immunoassay by use of composite nanoparticles as signal labels. TrAC-Trend. Anal. Chem..

[18] Gao Z., Ye H., Tang D., Tao J., Habibi S., Minerick A., Tang D., Xia X. (2017). Platinum-Decorated Gold Nanoparticles with Dual Functionalities for Ultrasensitive Colorimetric in Vitro Diagnostics. Nano Lett..

[19] Wei Z., Luciano K., Xia X. (2022). Catalytic Gold-Iridium Nanoparticles as Labels for Sensitive Colorimetric Lateral Flow Assay. ACS Nano.

[20] Rahbar M., Wu Y., Subramony J.A., Liu G. (2021). Sensitive Colorimetric Detection of Interleukin-6 via Lateral Flow Assay Incorporated Silver Amplification Method. Front. Bioeng. Biotechnol..

[21] Panferov V.G., Safenkova I.V., Varitsev Y.A., Drenova N.V., Kornev K.P., Zherdev A.V., Dzantiev B.B. (2016). Development of the Sensitive Lateral Flow Immunoassay with Silver Enhancement for the Detection of *Ralstonia solanacearum* in Potato Tubers. Talanta.

[22] Lei R., Arain H., Obaid M., Sabhnani N., Mohan C. (2022). Ultra-Sensitive and Semi-Quantitative Vertical Flow Assay for the Rapid Detection of Interleukin-6 in Inflammatory Diseases. Biosensors.

[23] Lei R., Wang D., Arain H., Mohan C. (2022). Design of Gold Nanoparticle Vertical Flow Assays for Point-of-Care Testing. Diagnostics.

[24] Jiang N., Ahmed R., Damayantharan M., Ünal B., Butt H., Yetisen A.K. (2019). Lateral and Vertical Flow Assays for Point-of-Care Diagnostics. Adv. Healthc. Mater..

[25] Han Q., Fan L., Liu X., Tang Y., Wang P., Shu Z., Zhang W., Zhu L. (2023). Lateral Flow Immunoassay Based on Quantum-Dot Nanobeads for Detection of Chloramphenicol in Aquatic Products. Molecules.

[26] Liu H., Liu Y., Zhou T., Zhou P., Li J., Deng A. (2022). Ultrasensitive and Specific Detection of Anticancer Drug 5-Fluorouracil in Blood Samples by a Surface-Enhanced Raman Scattering (SERS)-Based Lateral Flow Immunochromatographic Assay. Molecules.

[27] Hu J., Jiang Y.Z., Tang M., Wu L.L., Xie H.Y., Zhang Z.L., Pang D.W. (2019). Colorimetric-Fluorescent-Magnetic Nanosphere-Based Multimodal Assay Platform for Salmonella Detection. Anal. Chem..

[28] Zhu J., Guo G., Liu J., Li X., Yang X., Liu M., Fu C., Zeng J., Li J. (2024). One-pot synthesized Au@Pt nanostars-based lateral flow immunoassay for colorimetric and photothermal dual-mode detection of SARS-CoV-2 nucleocapsid antibody. Anal. Chim. Acta.

[29] Bradley Z., Coleman P.A., Courtney M.A., Fishlock S., McGrath J., Uniacke-Lowe T., Bhalla N., McLaughlin J.A., Hogan J., Hanrahan J.P. (2023). Effect of Selenium Nanoparticle Size on IL-6 Detection Sensitivity in a Lateral Flow Device. ACS Omega.

[30] Zhou W., Hu K.Q., Kwee S., Tang L., Wang Z.H., Xia J.F., Li X.J. (2020). Gold Nanoparticle Aggregation-Induced Quantitative Photothermal Biosensing Using a Thermometer: A Simple and Universal Biosensing Platform. Anal. Chem..

[31] Zhang D., Du S., Su S., Wang Y., Zhang H. (2019). Rapid detection method and portable device based on the photothermal effect of gold nanoparticles. Biosens. Bioelectron..

[32] Liang J., Wu L., Wang Y., Liang W., Hao Y., Tan M., He G., Lv D., Wang Z., Zeng T. (2023). SERS/photothermal-based dual-modal lateral flow immunoassays for sensitive and simultaneous antigen detection of respiratory viral infections. Sens. Actuators B Chem..

[33] Wang K., Liu X., Liang X., Jiang Y., Wen C.-Y., Zeng J. (2024). Near-Infrared Responsive Ag@Au Nanoplates with Exceptional Stability for Highly Sensitive Colorimetric and Photothermal Dual-Mode Lateral Flow Immunoassay. Anal. Chem..

[34] Wang Z., Wang M., Wang X., Hao Z., Han S., Wang T., Zhang H. (2023). Photothermal-based nanomaterials and photothermal-sensing: An overview. Biosens. Bioelectron..

[35] Xu S., Bai X., Wang L. (2018). Exploration of photothermal sensors based on photothermally responsive materials: A brief review. Inorg. Chem. Front..

[36] Zhang Y., Yang H.-J., Xu Z., Liu X., Zhou J., Qu X.-F., Wang W.-L., Feng Y., Peng C. (2023). An ultra-sensitive photothermal lateral flow immunoassay for 17β-estradiol in food samples. Food Chem..

[37] Zhang Y., Wang Z., Qu X., Zhou J., Yang H., Wang W., Yang C. (2023). A photothermal lateral flow immunoassay for zearalenone with high sensitivity and wide detection range. Sens. Actuators B Chem..

[38] Hao W., Huang Y., Wang L., Liang J., Yang S., Su L., Zhang X. (2023). Smartphone-Based Photothermal Lateral Flow Immunoassay Using Rhenium Diselenide Nanosheet. ACS Appl. Mater. Interfaces.

[39] Wen C.Y., Zhao L.J., Wang Y., Wang K., Li H.W., Li X., Zi M., Zeng J.B. (2023). Colorimetric and photothermal dual-mode lateral flow immunoassay based on Au-Fe_3_O_4_ multifunctional nanoparticles for detection of Salmonella typhimurium. Microchim. Acta.

[40] Guo G., Zhao T., Sun R., Song M., Liu H., Wang S., Li J., Zeng J. (2024). Au-Fe_3_O_4_ dumbbell-like nanoparticles based lateral flow immunoassay for colorimetric and photothermal dual-mode detection of SARS-CoV-2 spike protein. Chin. Chem. Lett..

[41] Moreau L.M., Schurman C.A., Kewalramani S., Shahjamali M.M., Mirkin C.A., Bedzyk M.J. (2017). How Ag Nanospheres Are Transformed into AgAu Nanocages. J. Am. Chem. Soc..

[42] Li X., Yu D., Li H., Sun R., Zhang Z., Zhao T., Guo G., Zeng J., Wen C.-Y. (2023). High-density Au nanoshells assembled onto Fe_3_O_4_ nanoclusters for integrated enrichment and photothermal/colorimetric dual-mode detection of SARS-CoV-2 nucleocapsid protein. Biosens. Bioelectron..

